# 2.5-Dimensional Parylene C micropore array with a large area and a high porosity for high-throughput particle and cell separation

**DOI:** 10.1038/s41378-018-0011-8

**Published:** 2018-06-18

**Authors:** Yaoping Liu, Han Xu, Wangzhi Dai, Haichao Li, Wei Wang

**Affiliations:** 1 Institute of Microelectronics, Peking University, Beijing, 100871 China; 2 Department of Respirology, No. 1 Hospital of Peking University, Beijing, 100034 China; 3 National Key Laboratory of Science and Technology on Micro/Nano Fabrication, Beijing, 100871 China; 4 Innovation Center for Micro-Nano-electronics and Integrated Systems, Beijing, 100871 China

## Abstract

Large-area micropore arrays with a high porosity are in high demand because of their promising potential in liquid biopsy with a large volume of clinical sample. However, a micropore array with a large area and a high porosity faces a serious mechanical strength challenge. The filtration membrane may undergo large deformation at a high filtration throughput, which will decrease its size separation accuracy. In this work, a keyhole-free Parylene molding process has been developed to prepare a large (>20 mm × 20 mm) filtration membrane containing a 2.5-dimensional (2.5D) micropore array with an ultra-high porosity (up to 91.37% with designed pore diameter/space of 100 μm/4 μm). The notation 2.5D indicates that the large area and the relatively small thickness (approximately 10 μm) of the fabricated membranes represent 2D properties, while the large thickness-to-width ratio (10 μm/ < 4 μm) of the spaces between the adjacent pores corresponds to a local 3D feature. The large area and high porosity of the micropore array achieved filtration with a throughput up to 180 mL/min (PBS solution) simply driven by gravity. Meanwhile, the high mechanical strength, benefiting from the 2.5D structure of the micropore array, ensured a negligible pore size variation during the high-throughput filtration, thereby enabling high size resolution separation, which was proven by single-layer and multi-layer filtrations for particle separation. Furthermore, as a preliminary demonstration, the prepared 2.5-dimensional Parylene C micropore array was implemented as an efficient filter for rare cancer cell separation from a large volume, approximately 10 cells in 10 mL PBS and undiluted urine, with high recovery rates of 87 ± 13% and 56 ± 13%, respectively.

## Membranes: Steep walls speed up separations

Polymer films that detect cancer cells in blood or urine samples using size-based filtrations can benefit from a strategy for improving the strength and throughput rate of micropore arrays. Yaoping Liu from Peking University in China and colleagues first fabricated a silicon wafer of close-packed micropillars, and then coated this template with the polymer film Parylene C. The team used reactive ion etching to carve out a hexagonal array of microscale openings in the polymer with unique dimensions—smaller-than normal pore separations for high flow rates, and thickness more than twice the width of the sidewall to give exceptional mechanical stability. Experiments proved the new array structure could selectively trap particles ranging from microbeads to cancer cells with throughputs hundreds of times faster than conventional filtering devices.

## Introduction

The successful separation of rare cancer cells from a clinical sample is extremely important for liquid biopsies in precision medicine^1–11^. Among the developed techniques, filtration has been considered the most promising approach due to its capability of achieving a high throughput, usually approximately 1 mL/min^6–11^. Various filtration techniques have been developed in the past decade, but for the liquid biopsy of large-volume clinical samples, such as bronchoalveolar lavage fluid (~80 mL), urine (~100 mL), and pleural effusion (~500 mL), the present filtration throughput still needs further improvement.

The current state of the art of filtration-based liquid biopsy is summarized in Table 1. Polymer filtration membranes fabricated via a track etching method can be traced back to the 1960s^12,13^ and have been widely utilized in biological studies and clinical practice for cell enrichment^6,14^. A large area is achievable for these track-etched membranes. However, the porosity of these membranes is very low (less than 1%), and therefore they cannot be used for high-throughput liquid biopsy. The other strategy to prepare filtration membranes is the lithography-assisted microfabrication technique^15–25^. Wit et al.^15^ utilized deep reactive iron etching (DRIE) and potassium hydroxide etching approaches to produce Si micropore arrays with an area of 0.64 cm^2^ and a porosity of 10%. Adams et al.^16–18^ fabricated an SU-8 filtration membrane with micropore arrays distributed within a 9 mm circular area with a porosity of <12.5% and achieved a throughput of >1 mL/min. Yoon-Tae Kang et al.^19^ developed a tapered-slit SU-8 membrane with a porosity up to 11% to increase the high throughput of viable circulating tumor cell (CTC) isolation^19^. Hosokawa et al.^20,21^ fabricated microcavity-arrayed poly(ethylene terephthalate) (PET) and Ni membranes via laser drilling^20^ and photolithography-based electroforming^21^, respectively. Although the area of these microcavity array could be easily extended to >1 cm^2^, the porosity of their filtration membrane was less than 2.25%, so the throughput from the aforementioned microcavity arrays was still less than 0.2 mL/min.

**Table 1:** The previously reported filtering devices for CTC (peripheral blood)-based liquid biopsy

Ref. No.	Filtration structure	Diameter	Pore-to-pore space	Area	Porosity	Throughput (normalized to mL/min)	Motor of filtration
^14^	Track-etched micropores	7.5 or 6.5 μm	N/A (randomly distributed)	1 cm^2^	N/A	~1 mL/min	Aspiration created by a vacuum tube collector
^15^	Silicon micropore array	5 μm	14 μm	0.64 cm^2^	5.4%	~2.3 mL/min	Aspiration created by a pump
^16–18^	SU-8 micropore array	5-9 μm	N/A	<0.64 cm^2^	<12.5%	5 mL/min	Aspiration created by a pump
^19^	SU-8 tapered-slit membrane	6 µm × 30 µm	N/A	1 cm^2^	6.2%	5 mL/h	Aspiration created by a pump
		8 µm × 40 µm			11%	10 mL/h	
^20^	PET microcavity array	2 μm	60 μm	4 cm^2^	0.008%	0.18 mL/min	Aspiration created by a pump
^21^	Ni microcavity array	9 μm	60 μm	1 cm^2^	0.64%	0.2–1 mL/min	Aspiration created by a pump
^22^	PEGDA conical holes	5.5–8 μm	22–24.5 μm	0.81 cm^2^	3.25–5.88%	0.2 mL/min	Aspiration created by a pump
^23^	Parylene C microspring structures	4, 5, 6, 7 μm (gap width)	N/A	0.5 cm^2^	N/A	0.75 mL/min	Aspiration created by a pump
^24^	Parylene C micropore array	40 × 6 μm (rectangular)	N/A	0.36 cm^2^	18%	0.2 mL/min	Aspiration created by a pressure gauge
^25^	2D Parylene C micropore array (single-layer)	10 μm (circular)	10 μm	1 cm^2^	<5.6%	<0.1 mL/min	Manual injection by a syringe
		14 × 8 μm (rectangular)	12 μm				
^26^	3D Parylene C micropore arrays (double-layer)	9 μm (top membrane) 8 μm (bottom membrane)	11 μm (top membrane) 12 μm (bottom membrane)	1 cm^2^	<6.96%	2–3.3 mL/min	Manual injection by a syringe
^27^	3D palladium micropocket array	30 μm (upper layer) 8 μm (lower layer)	4 μm (upper layer) 26 μm (lower layer)	1 cm^2^	5.02%	2–2.5 mL/min	Aspiration created by an efflux control unit

Tang et al.^22^ fabricated a mechanically strong polyethylene (glycol) diacrylate (PEGDA) microfilter containing conical holes via ultraviolet-assisted molding, but its porosity remained very low (<5.88%), and the filtration throughput was consequently limited to only 0.2–2.0 mL/min. Harouaka et al.^23^ fabricated Parylene C microspring structures via simple photolithography, achieving a throughput of 0.75 mL/min. Xu et al.^24^ developed a rectangular-pore array Parylene C membrane and achieved a porosity of 18%, along with a throughput >0.2 mL/min. Zheng et al.^25^ fabricated a 1 cm^2^ Parylene C micropore-array membrane via photolithography-based patterning and direct etching of Parylene C. The edge-to-edge spaces between adjacent pores were large (>10 μm), and the porosity was small (<5.6% with the large supporting structures considered), resulting in a filtration throughput of approximately 0.1 mL/min ^25^.

In short, owing to the fabrication difficulties and mechanical limitations, the previously reported micropore-array filtration structures^15–22,24–28^ usually had a relatively large supporting space (edge-to-edge distance) between the adjacent micropores and thereby also had a low porosity (the maximum reported was 18%^24^), which led to a limited, although high enough for CTC-based liquid biopsy, filtration throughput (<5 mL/min). Moreover, the low porosity caused a large filtration resistance and thus a large pressure during filtration, which seriously damaged the cells trapped in the micropores. Zheng et al.^26^ and Zhou et al.^27^ further developed a 3-dimensional (3D) double-layered Parylene C membrane filter to decrease the stress experienced by the trapped cells. In this filter, the space between the adjacent pores on the bottom membrane supported the cells trapped on the top membrane and thus relieved the stress on the cells trapped in the top membrane. Yusa et al.^28^ also developed a 3D palladium filter containing micropocket arrays with two different pore sizes in the upper and lower layers by using sequential lithography and electroforming techniques. Although the produced device exhibited a large area of 1 cm^2^, it still suffered from a relatively low throughput (2.0–2.5 mL/min) subject to the low porosity of <5.5%. Moreover, the non-transparency of palladium degraded its applicability in cellular research studies.

Parylene C is a popular polymer material in the field of biomedical microelectromechanical systems due to its high biocompatibility, optical transparency, and compatibility with the microfabrication processes. Due to the limited anisotropic etching capability of Parylene C^29^, it is difficult to obtain a micropore array with a small spaces (high porosity) and steep sidewall profiles (precision and uniformity of the pore size) by direct patterning and dry etching. A Parylene C molding procedure was developed by Suzuki et al.^30^ and Kuo et al.^31^ to fabricate suspended microsprings and microbeams with high aspect ratios and relatively large feature sizes (>10 μm). By using the Parylene C molding technique, our previous work^32^ reported a large, close-packed hexagonal micropore array with an ultra-high porosity and good size controllability. Cell separation by the prepared Parylene C micropore array with high throughput (~2 mL/min) and recovery rate (up to 96.5%) was preliminarily demonstrated^33^. However, in the previous work, the mechanical properties of the filtration membrane and the separation precision were not studied, which are key parameters for robust and reliable liquid biopsy. In this work, a modified molding process was developed to prepare a keyhole-free 2.5-dimensional (2.5D) Parylene C micropore array. The notation 2.5D indicates that the large area and the relatively small thickness (approximately 10 μm) of the fabricated membranes represent 2D properties, while the large thickness-to-width ratio (10 μm/ < 4 μm) of the spaces (edge-to-edge distances) between the adjacent pores corresponds to a local 3D feature. A chromatic confocal imaging (CCI) platform^34,35^ was applied to characterize the mechanical properties of the prepared micropore array filtration membranes. The separation precision of the filtration membrane was scrutinized through single-layer and multi-layer filtrations of rigid particles. Samples of 10 mL phosphate-buffered saline (PBS) and undiluted healthy urine containing ~10 spiked bladder epithelial cancer cells were filtered to demonstrate the high recovery rate of the prepared 2.5D Parylene C micropore array.

## Materials and methods

### Design of the micropore-array filtration membrane

To maximize the porosity of the filtration membrane, a close-packed, hexagonal micropore array was designed. Different pore diameters/spaces were investigated to optimize the filtration performance, cases 1-4, as listed in Table 2. In addition to the edge-to-edge space, defined as *s*, there are two parameters that describe the feature size of the hexagonal pore, *d* and *d'*, which correspond to the diagonal and edge-to-edge lengths, respectively. In this work, the diagonal lengths were designed to be 8, 10, 12, 15, and 100 μm, and two edge-to-edge spaces (2 and 4 μm) were tested. For each filtration membrane, the overall size was 20 mm × 20 mm with an effective filtration area of >13 mm × 13 mm. The prepared micropore array featured a 2.5D property: the large area (20 mm × 20 mm) and small thickness (~10 μm) of the filtration membrane represents a 2D geometry, while the high thickness-to-width ratio (10 μm/ < 4 μm) of the spaces between the adjacent micropores corresponds to a local 3D feature.

**Table 2:** The parameters of 2.5D Parylene C micropore-arrayed membrane for filtration investigation and optimization

Case no.	Design value in mask (μm)			Measured value after preparation (μm)			Porosity (%)
	*d*	*d’*	*s*	*d*	*d’*	*s*	
1	8	6.93	4	7.51 ± 0.13	6.50 ± 0.11	4.46 ± 0.09	40.19
2	10	8.66	4	9.13 ± 0.09	7.90 ± 0.08	4.69 ± 0.24	46.79
3	12	10.40	4	11.21 ± 0.11	9.71 ± 0.10	4.77 ± 0.15	52.14
4	15	12.99	4	14.52 ± 0.20	12.57 ± 0.17	4.43 ± 0.06	58.46
5	12	10.40	2	11.42 ± 0.17	9.89 ± 0.15	2.46 ± 0.11	70.33
6	100	86.60	4	99.35 ± 0.50	86.04 ± 0.43	4.48 ± 0.11	91.37

### Fabrication process

**Fig. 1:**
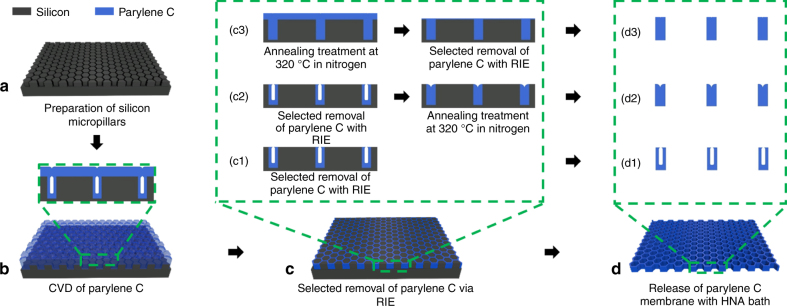
Schematic illustrations of the conventional and modified molding processes. Conventional molding process utilized for the fabrication of 2.5D micropore-array Parylene C filtration membranes without annealing (**a**, **b**, **c1**, **d1**) and a modified procedure that includes annealing treatment at 320 °C in nitrogen atmosphere for 2 h after (**a**, **b**, **c2**, **d2**) and before (**a**, **b**, **c3**, **d3**) the RIE stage

The fabrication procedure is schematically described in Fig. 1. First, a template containing the high-density, close-packed hexagonal micropillar array with a height of 10 μm on a 4-inch Si wafer was prepared via conventional photolithography patterning and DRIE (Fig. 1a). Second, a Parylene C layer with a specified thickness was deposited onto the Si template using a commercial Parylene deposition instrument (SCS, PDS2010; Fig. 1b). After that, reactive ion etching (RIE) of the deposited Parylene C layer was performed until the top of the silicon pillars was exposed (Fig. 1c1). An additive annealing at a temperature of 320 °C in a nitrogen atmosphere for 2 h before and after the RIE step was compared (see Fig. 1c2, c3, respectively). Finally, the prepared micropore-array Parylene C membrane was released from the Si substrate by soaking it in an HNA (HF:HNO3:HAc = 5:7:11, v/v) bath (Fig. 1d).

### Mechanical characterization

The mechanical properties of the fabricated 2.5D micropore-array Parylene C membranes were examined using the CCI-based platform described in detail elsewhere^34,35^. Briefly, a concentrated load was applied to the membrane surface, and the vertical displacement corresponding to the applied stress was measured and recorded. Subsequently, the theory of a circular plate with a large deflection by Timoshenko et al.^36^ was used to analyze the experimental data, and an equivalent Young’s modulus was calculated according to Eq. (1)^35^ to characterize the mechanical strength of the membrane.

Supposing the membrane was clamped at the edges and the concentrated load *q*_*c*_ was applied at the center of the membrane, the relationship between the deflection *d*_*v*_ at the center and the load can be expressed by Eq. (1):

1 $$\frac{{353 - 191\mu }}{{648(1 - \mu )}}Ehd_v^3 + \frac{{4Eh^3d_v}}{{3(1 - \mu )}} = \frac{{q_ca^2}}{\pi },$$

where *μ* represents the Poisson ratio of Parylene C, which is 0.5; *h* is the thickness of the membrane; *a* is the radius of the area of membrane; and *E* is the equivalent Young's modulus of the 2.5D micropore-array Parylene C membrane. By fitting the experimental data into Eq. (1), the equivalent Young's modulus of each membrane was extracted. Furthermore, the obtained equivalent Young’s modulus was used to predict the deformation of the 2.5D micropore-array membranes during the actual filtration process. A COMSOL Multiphysics model was established to calculate the hydraulic pressure and shear stress applied on the membrane. A laminar flow model with a constant flow rate boundary condition was built and solved. The average pressure and shear stress on the membranes were calculated at a certain filtration throughput (100–200 mL/min), which exactly covered the flow rates in our particle separation experiments (150.78 ± 5.41 mL/min for single-layer filtration and 118.44 ± 4.81–179.95 ± 10.12 mL/min for multi-layer filtration with a PBS solution). After that, the vertical displacement of the entire membrane was estimated from the calculated pressure and equivalent Young’s modulus using Eq. (2)^35^.

2 $$\frac{{23 - 9\mu }}{{252(1 - \mu )}}Ehd_v^3 + \frac{{2Eh^3d_v}}{{9(1 - \mu )}} = \frac{{q_ua^2}}{{24}},$$

where *q*_*u*_ represents the uniformly distributed load on the membrane, and *d*_*v*_ is the vertical displacement at the center of the membrane. Finally, the lateral size variation of a single micropore, δ*R*, was estimated based on the calculated displacement, *d*_*v*_, of the membrane using Eq. (3):

3 $${\mathrm{\delta R}} = \sqrt {{\mathrm{R}}^2 + d_V^2},$$

where *R* represents the original radius of the membrane, *δ* represents the change rate of the radius, as well as the pore size, and *R* is the diameter of the micropore.

### Particle separations via single-layer/multi-layer filtrations

To test the filtration performance of the present micropore-array filtration membranes, particles with varied diameters were chosen as the separation targets and went through single-layer or multi-layer filtrations.

**Fig. 2:**
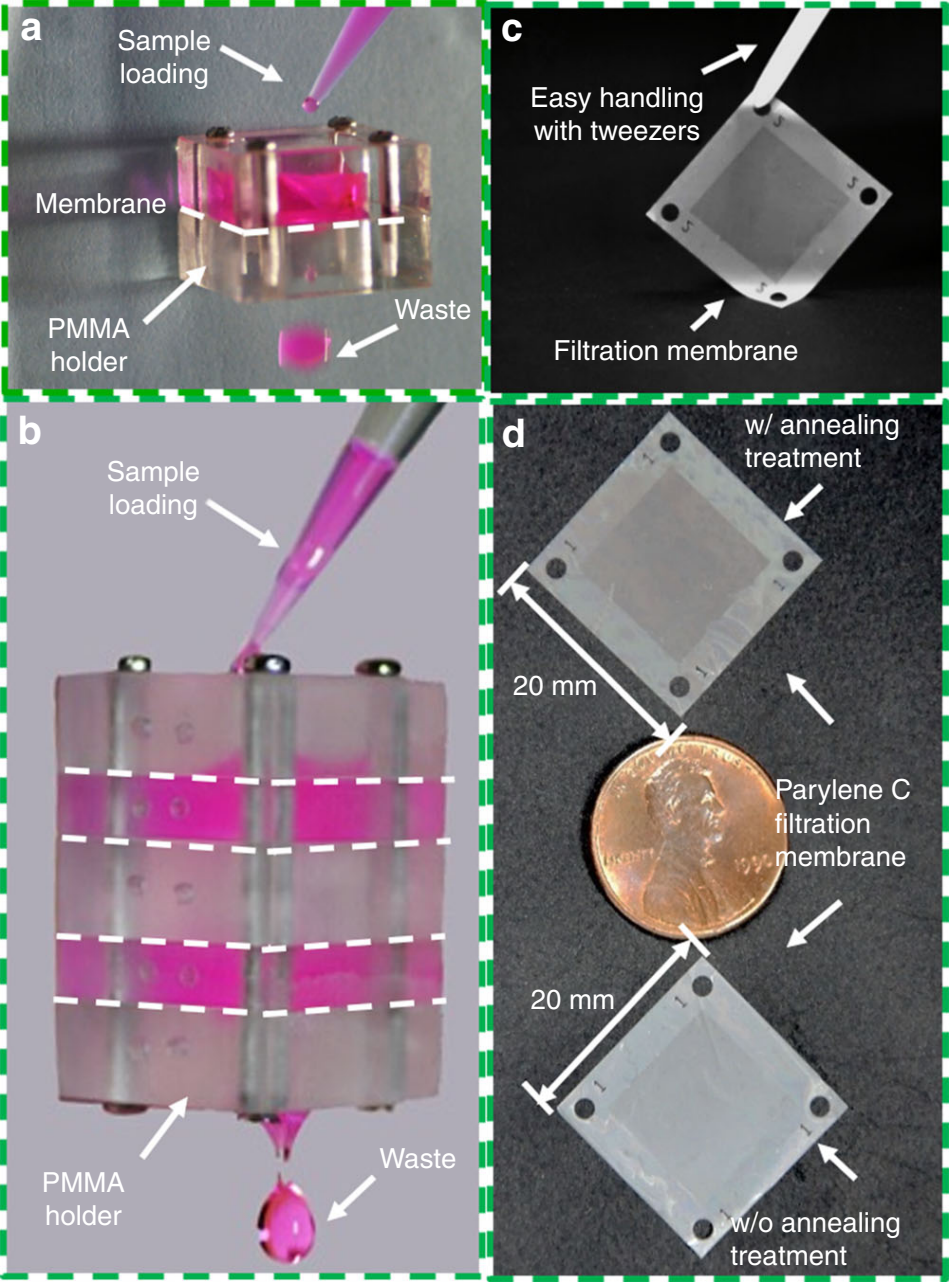
2.5D micropore-array Parylene C membrane and the filtration system. **a** A single membrane packaged for single-layer filtration. **b** An assembly with a homemade PMMA holder containing four membranes with different pore sizes/diameters for multi-layer filtration applications. **c** Easy handling of the fabricated filtration membrane with tweezers. **d** Photographs of the Parylene C membranes prepared with and without the annealing treatment

In the single-layer filtration test, a Parylene C membrane (case 3 in Table 2) was packaged with a homemade poly(methyl methacrylate) (PMMA) holder, as shown in Fig. 2a. The utilized sample loading procedure is schematically shown in Fig. 2a. The sample for separation was prepared via the addition of two monodispersed polystyrene (PS) particles (4000 Series Monosized Particles, Thermo Scientific, USA) with nominal diameters of 9 μm and 12 μm (8.1 × 10^4^/mL and 2.9 × 10^4^/mL, respectively) into 3.2 mL deionized (DI) water and well mixed on a vortex. After taking 200 μL out for a diameter measurement, the other 3 mL solution was loaded into the packaged single-layer filtration system (Fig. 2a). The throughout here was obtained based on the volume of the solution (3 mL DI water with 3.3 × 10^5^ particles), and the filtration time length was extracted from the recorded video of the whole filtration process. Then, the solution after filtration was collected and concentrated to 200 μL via centrifugation. The solutions containing particles before and after filtration were loaded (200 μL) into a high-throughput imaging flow cytometry system (FCM, Amnis®, ImageStream ^X^, Merck, Germany) to record particle images. Subsequently, the pictures were manually checked to screen the well-focused ones and measure the particle diameters. Meanwhile, the Parylene C membrane used in the filtration was disassembled from the PMMA holder and coated with a 3 nm thick Au layer to conduct scanning electron microscopy (SEM, JSM-7500F, JEOL, Japan) observations, which enabled a diameter check of particles trapped on the membrane.

In the multi-layer filtration, 4 pieces of 2.5D micropore-array Parylene C filtration membranes with different pore diameters/spaces were sequentially assembled using the homemade PMMA holder (Fig. 2b) in order to perform multiple-sized particle separation. The diameters/spaces of the filtration membranes arranged from the top to the bottom of the assembly were cases 4 to 1 in Table 2. A total of 2 mL DI water containing 4.5 × 10^6^ multi-dispersed PS particles with diameters ranging from 6 to 22 μm (BaseLine Chromtech Research Centre, Tianjin, China) was filtered through the assembled multi-layer filtration system. After filtration, the Parylene C membranes were disassembled and coated with a 3 nm thick Au layer to conduct SEM (JSM-7500F, JEOL, Japan) studies and obtain the sizes of the particle trapped on each membrane.

### Rare cell separation with single-layer filtration

Approximately 10 bladder epithelial cancer cells (T24) were spiked into 10 mL PBS and 10 mL undiluted midstream urine from a healthy volunteer. The addition of 10 T24 cells was realized via precise operation with a micropipette connecting to a microinjection pump (Fusion 200 series, Chemyx Inc., USA) under a stereomicroscope. The T24 cells (National Infrastructure of Cell Line Resource (Beijing)) were cultured with McCoy's 5a medium containing 10% Fetal bovine serum (FBS, Gibco, Thermo Fisher, USA), 2 mM Glutamine (Gibco, Thermo Fisher, USA), 1 mM sodium pyruvate (Gibco, Thermo Fisher, USA), 25 mM HEPES (Gibco, Thermo Fisher, USA), and 100 U/mL penicillin–streptomycin (Gibco, Thermo Fisher, USA). Before micropipette aspiration and transfer into the 10 mL PBS and urine, the adhered T24 cells (passages 10–20) were rinsed with 0.25% trypsin with 0.03% EDTA solution (Gibco, Thermo Fisher, USA), and then an additional 1 to 2 mL trypsin-EDTA solution was added into a T25 flask. The flask was allowed to sit at 37 °C until the T24 cells detached. The detached T24 cells were collected into a tube and centrifuged at 1000 rpm for 5 min. Then, the supernatant was disposed of, and the T24 cells were suspended into PBS and incubated with 5 μm Cell Tracker Red/Green (Invitrogen, Thermo Fisher, USA) and 1 μg/mL Hoechst 33342 (Invitrogen, Thermo Fisher, USA) at room temperature for 30 min for pre-labeling. After incubation, the cells were washed with PBS 3 times and re-suspended into the aforementioned McCoy's 5a medium for the micropipette operation. The 10 mL PBS and undiluted urine with spiked T24 cells were filtered following the same operation as particle separation through the micropore-array membrane with diameters/spaces of case 2 in Table 2. After filtration, the membranes were disassembled from the PMMA gadget, adhered to a slide and sealed with the ProLong antifade solution (ProLong® Diamond Antifade Mountant, Invitrogen, Thermo Fisher, USA) and a cover slip. The recovered cells on the membranes were observed and counted under a fluorescence microscope (CKX53, Olympus, Japan). For accuracy, every slide was checked 3 times. The cell separation experiments were repeated 5 times.

## Results and discussion

### 2.5D micropore-array Parylene C membranes

**Fig. 3:**
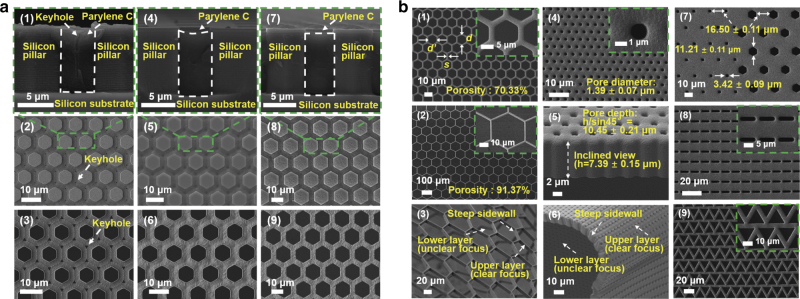
SEM images of the 2.5D micropore array Parylene C membranes. SEM images of the **a1** cross-sectional view and **a2** surface of Parylene C microstructures on the silicon template after RIE and **a3** released Parylene C membrane prepared via conventional molding. SEM images of the **a4** cross-sectional view and the **a5** surface of Parylene C microstructures on the silicon template and **a6** released Parylene C membrane obtained after the RIE stage of the modified molding process. SEM images of the **a7** cross-sectional view and **a8** surface of Parylene C microstructures on the silicon template and **a9** released Parylene C membrane obtained before the RIE stage of the modified molding process. **b** Membranes with diameters/spaces of cases 5 (**b1**) and 6 (**b2**) in Table 2. **b3** Image showing the steep sidewall profiles and excellent flexibility properties of the fabricated 2.5D micropore-array Parylene C membrane with high porosity. SEM images of the **b4** surface and **b5** inclined cross-sectional view of the Parylene C membrane with an aspect ratio of approximately 7.5. **b6** Image showing the steep sidewall profiles and excellent flexibility properties of the fabricated 2.5D micropore-array Parylene C membrane with a high aspect ratio. **b7** A combination of the hexagonal and square micropores. SEM images of the Parylene C membranes with a rectangular pattern (**b8**) and a triangle pattern (**b9**)

Figure 3a1, a2 shows the SEM images of the cross-sectional and top views of the Parylene C microstructures in the trenches between the adjacent silicon pillars, respectively, which were obtained after RIE and before the release stage of the molding process. Keyhole formation can be clearly observed on the top surface of the released membrane displayed in Fig. 3a3. Keyhole formation was unavoidable in the conventional molding process and has been well studied in the chemical vapor deposition field^30,31,37^. Although some attempts to reduce the size of the produced keyholes using etching/deposition cycling processes have been made, they were time consuming and complicated^30,31,37^. Herein, we proposed a simple annealing procedure, which was conducted at a temperature of 320 °C in a nitrogen atmosphere for 2 h, for keyhole removal. Figure 3a4–a6, a7–a9 contains the SEM images of the membrane subjected to annealing at a temperature of 320 °C in a nitrogen atmosphere for 2 h obtained after and before RIE, respectively. They show that all keyholes have been effectively removed by the annealing treatment because of the reflow of Parylene C.

The SEM image of the cross-sectional view of the Parylene C microstructure depicted in Fig. 3a4 exhibits a concave surface after keyhole removal during the annealing treatment of the modified molding process performed after RIE. In contrast, the cross-section of the Parylene C microstructure obtained with the annealing treatment conducted before RIE (Fig. 3a6) shows a flat surface. Figure 3a5, a8, a6, a9 displays the Parylene C microstructures on the silicon template after RIE and release of the 2.5D micropore-array Parylene C membranes. The surface topographies depicted in Fig. 3a8, a9 were smoother than those displayed in Fig. 3a5, a6. The surface profiles described in Fig. 3a7–a9 were better than those depicted in Fig. 3a4–a6, indicating that the annealing treatment conducted before RIE was more efficient than that performed after RIE during the preparation of the Parylene C membrane. The simple but efficient annealing treatment removed keyholes successfully and presented more stable and reproducible morphologies, which ensured higher reliability for practical applications.

The produced membranes were also annealed at 290 °C in a nitrogen atmosphere for 2 h, which corresponded to the highest reported working temperature for Parylene C in this work. However, the keyholes were nearly unchanged because 290 °C annealing did not cause the reflow of Parylene C. After annealing at 320 °C in a nitrogen atmosphere for 2 h, Parylene C lost approximately 5% of its original weight (according to the linear extrapolation from the data reported by Cao et al.^38^), while the positions of the X-ray diffraction peaks remained almost the same, indicating no significant changes in the chemical structure during the treatment. In addition, Metzen et al.^39^ investigated the chemical structure of annealed Parylene C via Fourier transform infrared spectroscopy and showed the absence of any changes observed after annealing for 3 h at 300 °C in a nitrogen atmosphere (two reduced bands were detected only until after 3 h of treatment at 350 °C). Thus, the annealing temperature of 320 °C is an appropriate option for keyhole removal while ensuring the absence of obvious weight losses or chemical structure changes. Therefore, all annealing treatments were performed at 320 °C in a nitrogen atmosphere for 2 h in this study, unless specified otherwise.

As shown in Fig. 2c, the fabricated 2.5D micropore-array Parylene C membrane can be easily handled with tweezers. Figure 2d displays two large (20 mm × 20 mm) filtration membranes prepared with and without annealing, which indicate unnoticeable differences in their physical appearance (including transparency).

Figure 3b1 shows the existence of two parameters describing the diameters of hexagonal micropores, *d* and *d’*, which correspond to the diagonal and edge-to-edge lengths, respectively. The pore diameter mentioned in this work represents the diagonal length *d* (unless specified otherwise). In particular, Fig. 3b1 shows the SEM image of a typical fabricated 2.5D micropore-array membrane with pore diameters/spaces of case 5 in Table 2 and a thickness-to-width ratio of the spaces between adjacent pores of 4.64. Figure 3b2 displays the membrane with pore diameters/spaces of case 6 in Table 2 and a porosity of up to 91.37%, which was the highest value obtained for the membranes fabricated in this study. Additionally, the so-prepared membrane exhibited steep sidewall profiles and excellent flexibility, as shown in Fig. 3b3. Figure 3b4 shows the Parylene C membrane fabricated with micropore diameters of 1.39 ± 0.07 μm and a depth of 10.45 ± 0.21 μm, as depicted in Fig. 3b5, which reveals an aspect ratio up to 7.5. The image depicted in Fig. 3b6 reveals that the membrane with a high ratio aspect presents steep sidewall profiles and excellent flexibility similar to the membrane with high porosities. Square micropores with a diameter of 3.42 ± 0.09 μm and hexagonal micropores with a diameter of 11.21 ± 0.11 μm, featuring the same space of 16.50 ± 0.11 μm, can be prepared on the same membrane, as shown in Fig. 3b7. In addition to the close-packed hexagonal array, other micropore patterns were also produced to show the capability of the present Parylene C molding process, and their corresponding SEM images are shown in Fig. 3b8, b9.

### Throughput test

**Fig. 4:**
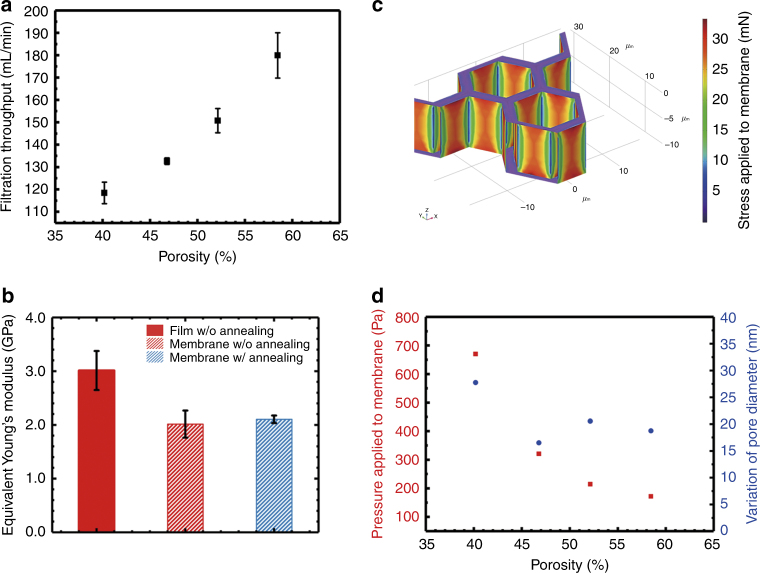
Calculation and simulation for the mechanical characterization of the 2.5D micropore array Parylene C membranes. **a** The measured filtration throughput of PBS driven by gravity with the prepared 2.5D micropore-array Parylene C membranes of different porosities, cases 1–4 in Table 2. **b** Equivalent Young’s modulus values obtained for the flat non-porous Parylene C film and 2.5D micropore-array Parylene C membranes with pore diameters/spaces of case 2 in Table 2, which were prepared with and without annealing treatment at a temperature of 320 °C in a nitrogen atmosphere for 2 h. **c** Numerical simulation of hydraulic pressure and shear stress. **d** Calculated pressures applied to the 2.5D micropore-array membranes (left *y*-axis, red) with different porosities and the corresponding micropore size variations (right *y*-axis, blue) observed during the actual filtration process

Owing to the high porosity of the 2.5D micropore-array Parylene C membrane, a high throughput of up to 180 mL/min (obtained at a porosity of 58.46% with a PBS solution) filtration for an aqueous (PBS was used in this study) solution simply driven by gravity was achieved, as shown in Fig. 4a. The filtration throughput (>3 experiments for each) obtained by the present 2.5D Parylene C micropore array is the highest reported data to the best of the authors’ knowledge, which mainly benefited from the high porosity and the large filtration area.

### Mechanical characterization

The mechanical behavior of circular plates with large deflections was theoretically described by Timoshenko et al.^33^ The obtained results were used in this study to estimate the equivalent Young’s modulus values from the experimentally measured vertical displacements under concentrated loads, as described in Eq. (1). The Young’s modulus of the flat Parylene C film (which served as the system control and was used for calibration purposes) was measured as 3.01 ± 0.36 GPa (calculated from the data of 5 tests), which is almost consistent with the results of previous studies (2.76 GPa, data from the datasheet of SCS^40^) and confirms the reliability of the present measurement setup and data extraction procedure. The equivalent Young’s modulus values of the 2.5D micropore-array Parylene C membranes with pore diameters/spaces of case 2 in Table 2 prepared with and without annealing treatment are compared in Fig. 4b (>3 samples for each).

The Young’s modulus of the flat Parylene C film decreased after annealing^36,38^, while the equivalent Young’s modulus of the fabricated 2.5D micropore-array Parylene C membranes remained almost unchanged after the annealing treatment. The retained high mechanical strength of the micropore-array membranes most likely resulted from their 2.5D structures.

With the established COMSOL Multiphysics model, the hydraulic pressure and shear stresses applied on the membranes during filtration were numerically simulated (shown in Fig. 4c). Thereby, the equivalent pressures applied to the 2.5D micropore-array Parylene C membranes with different porosities and the corresponding micropore size variations during the actual filtration process at a throughput higher than 110 mL/min (Fig. 4a) are shown in Fig. 4d. The negligible micropore size variations (<40 nm) indicated that the present 2.5D micropore-array membranes exhibited a high mechanical strength during the high-throughput filtration, which ensured their good separation performance, i.e., a high size resolution. Moreover, the elongation of the 2.5D micropore-array Parylene C membranes observed at the filtration throughput of 180 mL/min (with PBS solution) was below 0.28%, which was much smaller than the previously reported elongation yield of Parylene C (2.9% for the as-deposited Parylene C, from the datasheet of SCS^40^). Therefore, the 2.5D micropore-array membranes underwent an elastic displacement during the filtration operation.

### Microbead separation via single-layer and multi-layer filtrations

**Fig. 5:**
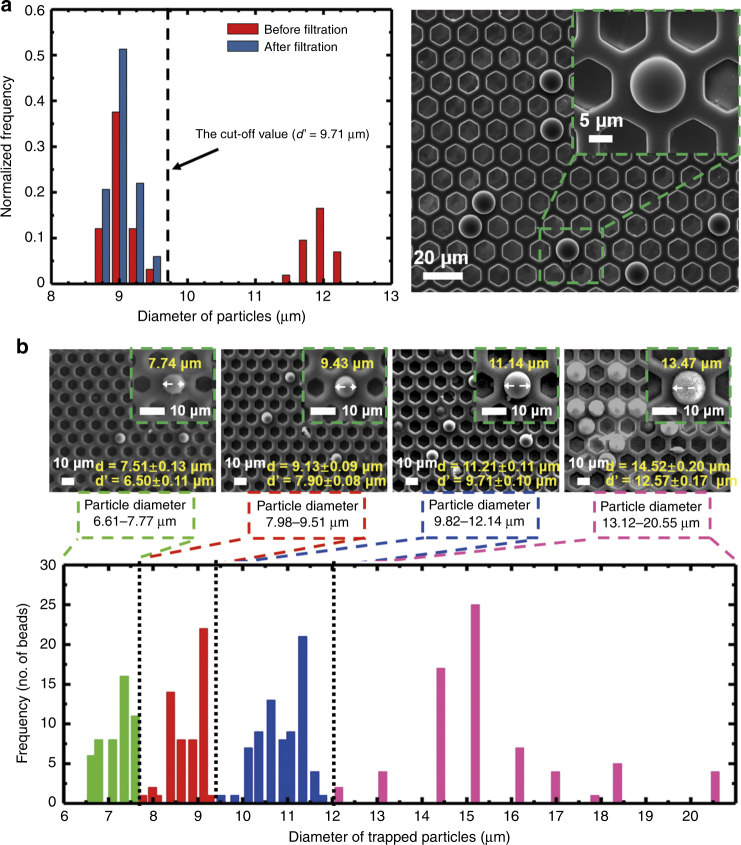
Particle separation results with the single-layer and multi-layer filtrations. **a** Particle separation of the single-layer filtration. Size distribution of the particles before and after filtration (the filtration membrane of case 3 in Table 2), and a typical SEM image of the particles trapped on the filtration membrane. **b** Particle separation of the multi-layer filtration. Typical SEM images of the particles trapped on the filtration membranes (cases 1 to 4), and size distribution of the particles trapped on the filtration membranes with different micropore diameters during the multi-layer filtration

To test the size-based separation performance of the present 2.5D micropore-array membranes, single-layer and multi-layer filtrations of microbeads with various diameters were carried out. In the single-layer filtration, monodispersed microbeads with different diameters were mixed as the loading sample. As shown in Fig. 5a, the size distribution of the microbeads before filtration showed two peaks centered at 9 μm and 12 μm. After filtration, only the peak centered at 9 μm remained, which revealed that only the microbeads with a diameter smaller than the edge-to-edge length *d*' (9.71 ± 0.20 μm) could go through the filtration membrane, while the microbeads of diameter larger than *d*' should have been trapped on the membrane, which was verified by the SEM image shown in Fig. 5a. In the multi-layer filtration, monodispersed microbeads with a diameter ranging from 6 μm to 22 μm were contained in the loading sample. The SEM images in Fig. 5b display the microbeads trapped on the Parylene C membranes with the edge-to-edge lengths of micropores, *d*', of cases 4-1 in Table 2. As indicated in Fig. 5b, the size distribution of the microbeads trapped on a membrane was exactly between the *d*’ of this membrane and that of the above membrane, which proved that the present filtration membrane had a good size resolution. The PS microbeads were rigid and could not deform; the good size resolution is hence attributed to the negligible size variation of the micropore during the filtration, even at a high throughput. This further confirmed the strong mechanical strength of the prepared 2.5D micropore-array Parylene C membrane. In addition, the non-specific adhesion of the microbeads to the supporting structure (space) between adjacent micropores was absent, which corresponded to a very high separation purity of filtration (close to 100%). The high mechanical strength of the present 2.5D micropore-array Parylene C membrane guaranteed the excellent filtration performance of rigid microbead separation. Nevertheless, in real liquid biopsy, the separation of live cells would require further optimization of the pore diameters, as cells could deform to go through micropores even smaller than their nominal diameter, which requires further investigation.

### Rare cell separation with single-layer filtration

**Fig. 6:**
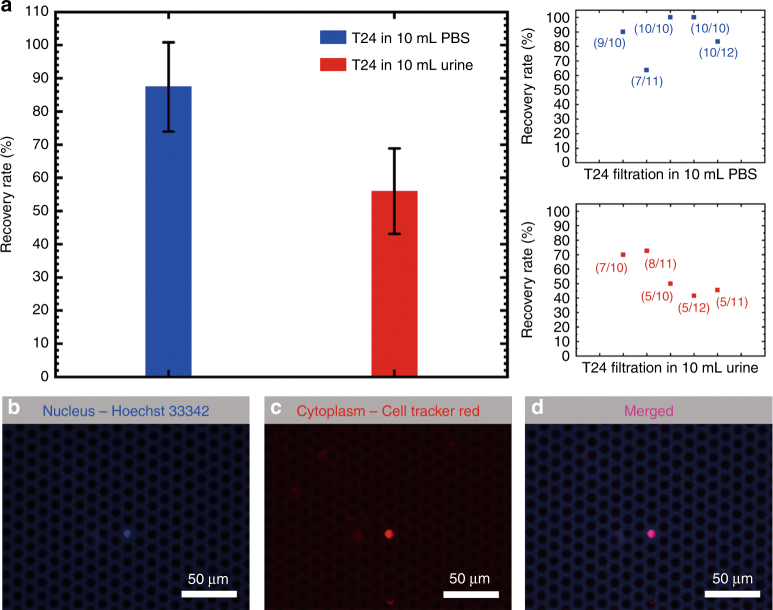
**a** Recovery rates for the T24 cells in 10 mL PBS and 10 mL undiluted urine by using a micropore array with a pore diameter of 9.13 ± 0.09 μm and a space of 4.69 ± 0.24 μm (case 2 in Table 2). **b**–**d** Captured T24 cells on the micropore array labeled with Hoechst 33342 (nucleus) and Cell Tracker Red (cytoplasm), and the merged image

The size of the T24 cells used in the present work was measured as 13.5 ± 0.84 μm (ranging from 8 μm to 20 μm) under the microscope in PBS suspension. Therefore, the Parylene C membrane with a pore edge-to-edge length of 7.90 ± 0.08 μm (case 2 in Table 2) was used for cell separation. The throughputs of the cell separation from PBS and undiluted urine by this micropore array were measured. For the PBS solution, the throughput was 133 mL/min, the same as that shown in Fig. 4a, while for the undiluted urine, the throughput dropped to 100 mL/min because of the large number of background cells but was still high enough to run a 10 mL sample in 6 s. As shown in Fig. 6a, 87 ± 13% and 56 ± 13% of spiked T24 cells (approximately 10 cells in each test) were successfully captured from the PBS and undiluted urine, respectively. Figure 6b–d shows the captured T24 cells on the micropore array labeled with Hoechst 33342 (nucleus) and Cell Tracker Red (cytoplasm) and the merged image.

## Conclusions

In this study, a modified Parylene molding process was developed for the preparation of large-area (>20 mm × 20 mm) 2.5D micropore-array membranes with ultra-high porosities (up to 91.37% with the designed pore diameter/space of 100 μm/4 μm). The keyholes that formed in the Parylene structures, unavoidable during the conventional molding process, were successfully removed via a simple and effective annealing treatment at 320 °C in a nitrogen atmosphere for 2 h. Owing to the high porosity of the prepared 2.5D micropore-array membrane, a high throughput up to 180 mL/min (obtained at the porosity of 58.46% with PBS solution) for an aqueous solution was successfully achieved simply driven by gravity. The high mechanical strength of the fabricated 2.5D micropore-array membrane ensured a negligible micropore size variation during the high-throughput filtration and thus resulted in a high size resolution in single-layer and multi-layer particle separations. The precise filtration capability ensured high recovery rate cell separations from a large volume of PBS or undiluted urine. The preliminary experimental results showed that by using a 7.90 ± 0.08 μm-sized micropore array, 87 ± 13% and 56 ± 13% of spiked T24 cells (approximately 10 cells in each test) were successfully captured from 10 mL PBS and undiluted urine, respectively. The throughputs were 133 mL/min for PBS and 100 mL/min for undiluted urine. This result indicated that the present 2.5D micropore-array membrane has promising potential in fulfilling high-throughput, high recovery rate liquid biopsy of large-volume clinical samples.

## References

[1] Arya SK, Lim B, Rahman AR (2013). Enrichment, detection and clinical significance of circulating tumor cells. Lab. Chip..

[2] Shields CW, Reyes CD, López GP (2011). Microfluidic cell sorting: a review of the advances in the separation of cells from debulking to rare cell isolation. Lab. Chip..

[3] Low WS, Abas WABW (2015). Benchtop technologies for circulating tumor cells separation based on biophysical properties. Biomed. Res. Int..

[4] Pesta M (2015). May CTC technologies promote better cancer management?. EPMA J..

[5] Alix-Panabières C, Pante K (2014). Technologies for detection of circulating tumor cells: facts and vision. Lab. Chip..

[6] Dolfus C (2015). Circulating tumor cell isolation: the assets of filtration methods with polycarbonate track-etched filters. Chin. J. Cancer Res..

[7] Jin C (2014). Technologies for label-free separation of circulating tumor cells: from historical foundations to recent developments. Lab. Chip..

[8] Yu L (2013). Advances of lab-on-a-chip in isolation, detection and post-processing of circulating tumour cells. Lab. Chip..

[9] Li P (2013). Probing circulating tumor cells in microfluidics. Lab. Chip..

[10] Harouaka RA, Nisic M, Zheng SY (2013). Circulating tumor cell enrichment based on physical properties. J. Lab. Autom..

[11] Chen J, Li J, Sun Y (2012). Microfluidic approaches for cancer cell detection, characterization, and separation. Lab. Chip..

[12] Fleischer RL (1964). Fission-track ages and track-annealing behavior of some micas. Science.

[13] Fleischer RL (1972). Particle track etching. Science.

[14] Desitter I (2011). A new device for rapid isolation by size and characterization of rare circulating tumor cells. Anticancer. Res..

[15] Wit SD (2015). The detection of EpCAM+ and EpCAM– circulating tumor cells. Sci. Rep..

[16] Adams DL (2013). The systematic study of circulating tumor cell isolation using lithographic microfilters. RSC Adv..

[17] Adams DL (2015). Cytometric characterization of circulating tumor cells captured by microfiltration and their correlation to the CellSearch(?) CTC test. Cytom. Part A.

[18] Adams DL (2016). Precision microfilters as an all in one system for multiplex analysis of circulating tumor cells. RSC Adv..

[19] Kang YT (2015). Tapered-slit membrane filters for high-throughput viable circulating tumor cell isolation. Biomed. Micro..

[20] Hosokawa M (2009). High-density microcavity array for cell detection: single-cell analysis of hematopoietic stem cells in peripheral blood mononuclear cells. Anal. Chem..

[21] Hosokawa M (2010). Size-selective microcavity array for rapid and efficient detection of circulating tumor cells. Anal. Chem..

[22] Tang Y (2014). Microfluidic device with integrated microfilter of conical-shaped holes for high efficiency and high purity capture of circulating tumor cells. Sci. Rep..

[23] Harouaka RA (2014). Flexible micro spring array device for high-throughput enrichment of viable circulating tumor cells. Clin. Chem..

[24] Xu T (2010). A cancer detection platform which measures telomerase activity from live circulating tumor cells captured on a microfilter. Cancer Res..

[25] Zheng S (2007). Membrane microfilter device for selective capture, electrolysis and genomic analysis of human circulating tumor cells. J. Chromatogr. A.

[26] Zheng S (2011). 3D microfilter device for viable circulating tumor cell (CTC) enrichment from blood. Biomed. Micro..

[27] Zhou MD (2014). Separable bilayer microfiltration device for viable label-free enrichment of circulating tumour cells. Sci. Report.

[28] Yusa A (2014). Development of a new rapid isolation device for circulating tumor cells (CTCs) Using 3D palladium filter and its application for genetic analysis. PLoS One.

[29] Meng E, Li PY, Tai YC (2008). Plasma removal of Parylene C. J. Micromech. Microeng..

[30] Suzuki Y, Tai YC (2006). Micromachined high-aspect-ratio parylene spring and its application to low-frequency accelerometers. J. Micro. Syst..

[31] Wen-Cheng K, Chen CW (2014). Fabrication suspended high-aspect-ratio parylene structures for large displacement requirements. Int. J. Autom. Smart Technol..

[32] Liu Y. et al. Filtration membrane with ultra-high porosity and pore size controllability fabricated by parylene C molding technique for targeted cell separation from bronchoalveolar lavage fluid (BALF). *Proc. 18th Int. Conf. on Solid-State Sensors, Actuators and Microsystems (Transducers 2015*) 1767–1769 (Anchorage, 2015).

[33] Liu Y. et al. Highly precise and efficient cell separation with parylene C micropore arrayed filtration membrane. *Proc. 19th Int. Conf. on Miniaturized Systems for Chemistry and Life Sciences (microTAS 2015)* 389 (Gyeongju, 2015).

[34] Dai W. et al. Chromatic confocal imaging based mechanical test platform for micro porous membrane. *Proc. 13th IEEE Int. Conf. on Solid-State and Integrated Circuit Technology (ICSICT 2016)* 16-6 (Hangzhou, 2016).

[35] Dai W. et al. Mechanical strength of 2.5D Parylene C micropore-arrayed filtration membrane. *19th International Conference on Solid-State Sensors, Actuators and Microsystems (TRANSDUCERS 2017)* 1215 (Kaohsiung, 2017).

[36] S. Timoshenko, S. Woinowsky-Krieger. *Theory of Plates and Shells* (McGraw-Hill, New York, 1959).

[37] Lei Y (2009). A parylene-filled-trench technique for thermal isolation in silicon-based microdevices. J. Micromech. Microeng..

[38] Cao Q (2008). Thermal decomposition of Parylene C film. J. Mater. Sci. Eng..

[39] von Metzen RP, Stieglitz T (2013). The effects of annealing on mechanical, chemical, and physical properties and structural stability of Parylene C. Biomed. Micro..

[40] Specialty Coating Systems, Inc. *SCS PARYLENE PROPERTIES*. Available at https://scscoatings.com/wp-content/uploads/2017/09/02-SCS-Parylene-Properties-1016.pdf.

